# Cell-free RNA profiling uncovers non-canonical circulating D2 transcript elevation in Bladder Cancer plasma

**DOI:** 10.1016/j.jlb.2025.100454

**Published:** 2026-01-03

**Authors:** Annarita Nappi, Felice Crocetto, Paolo Conforti, Serena Sagliocchi, Annunziata Gaetana Cicatiello, Federica Restolfer, Lucia Acampora, Silvia Del Mastro, Rosa Sirica, Lorenzo Spirito, Francesco Del Giudice, Roberto La Rocca, Daniela Terracciano, Monica Dentice, Caterina Miro

**Affiliations:** a Department of Clinical Medicine and Surgery, University of Naples “Federico II”, 80131, Naples, Italy; b Department of Neurosciences, Reproductive Sciences and Odontostomatology, University of Naples “Federico II”, 80131, Naples, Italy; c Department of Translational Medical Sciences, University of Naples “Federico II”, 80131, Naples, Italy; d Department of Women, Child and General and Specialized Surgery, University of the Studies of the Campania “L. Vanvitelli”, Naples, Italy; e ^D^epartment of Maternal Infant and Urologic Sciences, University La Sapienza, Rome, Italy; f ^I^nterdepartmental Center for Advances in Robotic Surgery, University of Naples “Federico II”, 80131, Naples, Italy; g CEINGE – Biotecnologie Avanzate S.c.a.r.l., 80131, Naples, Italy

**Keywords:** Thyroid hormones, Deiodinases, Bladder cancer, Liquid biopsy, Circulating biomarkers

## Abstract

**Background:**

D2 overexpression has emerged as a recurrent molecular feature across multiple cutaneous malignancies, where it contributes to aberrant Thyroid Hormone (TH) activation and tumor-associated metabolic reprogramming. Liquid biopsy approaches based on circulating cell-free RNA (cfRNA) is emerging as non-invasive strategy to profile gene expression alterations and support dynamic monitoring of transcriptional changes during disease progression.

**Methods:**

We analyzed 54 plasma samples from patients with BLadder CAncer (BLCA) alongside an equivalent cohort of healthy control individuals. Circulating D2 transcripts were quantified after RNA isolation using a modified phenol-chloroform extraction protocol adapted for low-input plasma samples to maximize retrieval of circulating RNA.

**Results:**

D2 transcripts were readily detectable in plasma and showed significantly higher levels in BLCA patients compared with healthy controls. Circulating expression of classical urothelial markers GATA3 and UPK3A, as well as Epithelial-to-Mesenchymal Transition (EMT)-related genes (E-Cadherin, N-Cadherin, Vimentin), was likewise increased in BLCA plasma. However, correlation analyses revealed that D2 expression varied independently from GATA3 and UPK3A across both tumor and non-tumor groups.

**Conclusions:**

These findings demonstrate that D2 is detectably elevated in the circulation of BLCA patients and captures tumor-associated transcriptional alterations that are independent of established urothelial markers. The distinct, non-redundant behavior of circulating D2 supports its potential value as a complementary biomarker for minimally invasive molecular profiling of BLCA. Further studies are required to define its diagnostic performance and clinical applicability.

## 1 Introduction

BLadder CAncer (BLCA) remains one of the most prevalent urological malignancies worldwide, with an estimated 550.000 new cases and approximately 165.000 deaths [1]. The disease encompasses two major clinical subtypes: Non-Muscle-Invasive Bladder Cancer (NMIBC), accounting for nearly 75 % of new diagnoses, and Muscle-Invasive Bladder Cancer (MIBC), which is associated with a worse prognosis [1].

Current diagnostic and surveillance strategies rely primarily on cystoscopy and urine cytology. While urine cytology offers high specificity (>90 %), its sensitivity for low-grade tumors remains unsatisfactory (<40 %), whereas cystoscopy, though accurate, is invasive, expensive, and associated with patient discomfort and potential complications [1,2]. Given that NMIBC is characterized by a recurrence rate of 50–70 %, patients often undergo lifelong invasive follow-up, which represents a substantial economic and psychological burden [1].

In this context, liquid biopsy has emerged as a promising, minimally invasive approach for the detection, monitoring, and molecular characterization of cancer. It refers to the analysis of tumor-derived components in body fluids such as blood or urine, including circulating cell-free RNA (cfRNA), Circulating Tumor Cells (CTCs), circulating tumor DNA (ctDNA) or cell-free DNA (cfDNA), Extracellular Vesicles (EVs), and tumor-associated proteins [[2], [3], [4], [5]]. For BLCA, urine represents a particularly attractive biofluid due to its direct contact with the urothelial lining, which enables continuous shedding of tumor-derived material [4,6].

The clinical utility of liquid biopsy in BLCA spans multiple applications: early detection and diagnosis, risk stratification, monitoring of recurrence (especially in NMIBC), detection of minimal residual disease after treatment (e.g., transurethral resection, intravesical therapy), assessment of therapeutic response, and guiding precision therapy in advanced disease.

Recent evidence indicates that Thyroid Hormone (TH) signaling contributes to tumor progression in several epithelial cancers, with its local activation controlled by iodothyronine deiodinases, particularly type II deiodinase (D2), which converts T4 into active T3. Notably, D2 expression is often elevated in aggressive tumors, where it is associated with metabolic reprogramming, stress adaptation, and epithelial-mesenchymal plasticity [[7], [8], [9], [10], [11], [12]]. From an endocrine-epidemiological perspective, thyroid dysfunction appears to modulate the risk and outcomes of BLCA. Several population-based analyses indicate that hyperthyroidism or elevated free T4 (fT4) levels are associated with an increased risk of bladder and other genitourinary cancers, whereas hypothyroid states correlate with slower tumor growth and more favorable outcomes. Consistently, a large retrospective cohort of over 30.000 patients reported a modest but significant increase in bladder-cancer risk among individuals with prior hyperthyroidism, while hypothyroid patients exhibited a reduced risk compared with euthyroid controls [[13], [14], [15]]. In addition, studies in thyroid cancer survivors treated with chronic Thyroid-Stimulating Hormone (TSH) suppression therapy revealed a higher rate of subsequent bladder neoplasms than in non-suppressed populations, supporting a potential pro-tumorigenic effect of prolonged TH exposure [15]. Importantly, pan-cancer transcriptomic analyses have reported D2 dysregulation in subsets of urothelial carcinomas as well, suggesting that altered TH metabolism may also influence BLCA biology [16]. Despite these insights, the relevance of D2 in BLCA has not been functionally explored, and its detectability in circulation remains unknown. Whether D2 behaves as a classical urothelial marker or instead reflects an independent biological axis, metabolic rewiring, cellular plasticity, or stress responses, has not been addressed. This gap is particularly relevant in the context of liquid biopsy, where biomarkers that provide non-redundant information could improve diagnostic accuracy and refine molecular profiling.

Building from a pan-cancer assessment of D2 expression [16], in this study we narrow the focus to BLCA as a whole and we investigate circulating D2 transcripts in plasma from BLCA patients and healthy controls. We compare D2 levels with established urothelial markers, GATA3 and UPK3A [[17], [18], [19], [20]] and EMT-related transcripts to determine whether D2 contributes a distinct circulating signature. By integrating endocrine-metabolic pathways with cfRNA-based profiling, we aim to assess the potential of D2 as a complementary biomarker for minimally invasive detection, supporting its utility as a liquid biopsy indicator. Embedding endocrine-metabolic factors into liquid-biopsy frameworks may open new opportunities for personalized monitoring and therapeutic targeting in this malignancy.

## 2 Materials and methods

### 2.1 Cohort and sample collection

This study enrolled adult patients (≥18 years) treated at the Urology Surgery Group of the University Hospital Center “Federico II”, Naples, Italy, between July 1, 2025, and October 31, 2025. Written informed consent was obtained from all participants prior to any procedure. The study was conducted in accordance with the Declaration of Helsinki, and all protocols were approved by the Ethics Committee of the University of Naples “Federico II” (Protocol number 118/20, May 7, 2020). Patients were stratified into two experimental groups: non-tumor and BLCA tumor. Individuals included in the BLCA group were histologically diagnosed with bladder cancer and underwent both tissue and liquid biopsy as part of routine clinical care. Furthermore, healthy adult volunteers donated peripheral blood samples, which served as the reference non-tumor control group for plasma analyses. Liquid biopsy analyses were performed on plasma-derived cfRNA isolated from peripheral blood samples. Details regarding patients’ age, sex, and histopathological features are synthetically summarized in Table 1 and detailed listed in Table 2. Blood samples were collected in 10 mL EDTA tubes before treatment. Plasma aliquots were isolated immediately following phlebotomy by centrifugation, frozen at −80 °C, and bio-banked. All downstream processing of frozen plasma from BLCA cases and healthy controls was performed concurrently, with samples interdigitated to minimize potential batch effects.

**Table 1:** Summary of the cohort enrolled in the study.

Variables	Healthy Control (n = 54)	BLCA Tumor (n = 54)	p-value
**Age (Mean ± Standard Deviation)**			
Age ≥18	54 (62.8 ± 15.0)	54 (67.6 ± 12.6)	0.06
Age <60	16 (43.8 ± 12.1)	14 (51.7 ± 10.6)	0.07
Age ≥60	38 (70.8 ± 6.5)	40 (73.4 ± 7.3)	0.09
**Sex (Mean ± Standard Deviation)**			
Female	11 (56.6 ± 16.4)	11 (60.3 ± 18.7)	0.63
Male	43 (64.3 ± 14.4)	43 (69.7 ± 9.9)	0.05

**Table 2:** Inclusive characterization of BLCA cohort demographics and tumor histopathology.

Group	Age	Sex
**BLCA patient 1**	74	male
Histopathological features	–	
Pathological stage	–	
**BLCA patient 2**	62	male
Histopathological features	High-grade papillary urothelial carcinoma, muscle-invasive	
Pathological stage	pT1bG2NxMx AJCC 2017 VIII ed.	
**BLCA patient 3**	72	male
Histopathological features	High-grade urothelial carcinoma extensively infiltrating the muscularis propria	
Pathological stage	pT2G4NoMx AJCC 2017 VIII ed.	
**BLCA patient 4**	78	male
Histopathological features	Urothelial carcinoma infiltrating only the superficial submucosal connective tissue; the fragments of muscularis propria present are free of neoplasia	
Pathological stage	pT1a G1NxMx AJCC 2017 VIII ed.	
**BLCA patient 5**	75	male
Histopathological features	High-grade papillary urothelial carcinoma, muscle-invasive	
Pathological stage	pT1eG3MxNx AJCC 2017 VIII ed.	
**BLCA patient 6**	68	male
Histopathological features	–	
Pathological stage	–	
**BLCA patient 7**	61	male
Histopathological features	Low-grade papillary urothelial carcinoma, muscle-invasive	
Pathological stage	pT1aG1NxMxAJCC 2017 VIII ed.	
**BLCA patient 8**	62	male
Histopathological features	Low-grade urothelial carcinoma (G1) not infiltrating the submucosal connective layer	
Pathological stage	pT1aG1NxMx AJCC 2017 VIII ed.	
**BLCA patient 9**	86	male
Histopathological features	–	
Pathological stage	–	
**BLCA patient 10**	67	male
Histopathological features	Low-grade papillary urothelial carcinoma not infiltrating the muscularis propria but extensively infiltrating the submucosal connective tissue	
Pathological stage	pT1bG1NoMx AJCC 2017 VIII ed.	
**BLCA patient 11**	72	male
Histopathological features	High-grade urothelial carcinoma extensively infiltrating the muscularis propria throughout its full thickness, with invasion of the prostatic parenchyma	
Pathological stage	pT3aG4NxMx AJCC 2017 VIII ed.	
**BLCA patient 12**	77	male
Histopathological features	High-grade urothelial carcinoma infiltrating the muscularis propria	
Pathological stage	pT2G3NxMx AJCC 2017 VIII ed.	
**BLCA patient 13**	79	male
Histopathological features	Hyperplastic urothelium	
Pathological stage	–	
**BLCA patient 14**	76	male
Histopathological features	–	
Pathological stage	–	
**BLCA patient 15**	56	male
Histopathological features	High-grade urothelial carcinoma extensively infiltrating the muscularis propria throughout its full thickness	
Pathological stage	pT2G4 NxMx AJCC 2017 VIII ed.	
**BLCA patient 16**	71	male
Histopathological features	High-grade papillary urothelial carcinoma infiltrating the muscularis propria throughout its full thickness, with extension into the prostatic parenchyma at the glandular base	
Pathological stage	pT2G3NxMx AJCC 2017 VIII ed.	
**BLCA patient 17**	21	female
Histopathological features	–	
Pathological stage	–	
**BLCA patient 18**	62	male
Histopathological features	–	
Pathological stage	–	
**BLCA patient 19**	56	male
Histopathological features	Papillary renal cell carcinoma	
Pathological stage	–	
**BLCA patient 20**	41	male
Histopathological features	High-grade papillary urothelial carcinoma infiltrating only the superficial submucosal connective tissue; fragments of muscularis propria present are free of disease	
Pathological stage	pT1aG3NxMx AJCC 2017 VIII ed.	
**BLCA patient 21**	59	male
Histopathological features	Hyperplastic urothelium	
Pathological stage	–	
**BLCA patient 22**	59	male
Histopathological features	–	
Pathological stage	–	
**BLCA patient 23**	71	male
Histopathological features	Fibropapilloma	
Pathological stage	–	
**BLCA patient 24**	65	female
Histopathological features	–	
Pathological stage	–	
**BLCA patient 25**	88	female
Histopathological features	Low-grade urothelial carcinoma not infiltrating the muscularis propria but infiltrating the submucosal connective layer	
Pathological stage	pT1bG1NxMx AJCC 2017 VIII ed.	
**BLCA patient 26**	77	male
Histopathological features	Low-grade urothelial carcinoma infiltrating the lamina propria and submucosal connective layer	
Pathological stage	pT1bG1NxMx AJCC 2017 VIII ed.	
**BLCA patient 27**	77	male
Histopathological features	–	
Pathological stage	–	
**BLCA patient 28**	74	male
Histopathological features	Low-grade urothelial carcinoma, non-invasive	
Pathological stage	pT1aG1NxMx AJCC 2017 VIII ed.	
**BLCA patient 29**	80	male
Histopathological features	High-grade urothelial carcinoma not infiltrating the lamina propria	
Pathological stage	pT1bG3NxMx AJCC 2017 VIII ed.	
**BLCA patient 30**	56	female
Histopathological features	Low-grade papillary urothelial carcinoma not infiltrating the lamina propria	
Pathological stage	pT1bG1NxMx AJCC 2017 VIII ed.	
**BLCA patient 31**	76	male
Histopathological features	High-grade urothelial carcinoma extensively infiltrating the muscularis propria, with invasion of the prostatic parenchyma	
Pathological stage	pT4G4N1Mx AJCC 2017 VIII ed.	
**BLCA patient 32**	45	female
Histopathological features	Urothelial hyperplasia with inflammatory features and nonspecific cytologic atypia	
Pathological stage	–	
**BLCA patient 33**	59	male
Histopathological features	–	
Pathological stage	–	
**BLCA patient 34**	46	female
Histopathological features	–	
Pathological stage	–	
**BLCA patient 35**	79	female
Histopathological features	High-grade carcinoma not infiltrating the lamina propria	
Pathological stage	pT1aG3NxMx AJCC 2017 VIII ed.	
**BLCA patient 36**	52	female
Histopathological features	High-grade chromophobe carcinoma (G4) extensively infiltrating the urothelial mucosa of the renal pelvis	
Pathological stage	pT3aG4NxMx sec AJCC 2017 VIII ed.	
**BLCA patient 37**	58	male
Histopathological features	High-grade urothelial carcinoma infiltrating only the initial portion of the muscularis propria	
Pathological stage	pT1bG3NxMx sec. AJCC 2017 VIII ed.	
**BLCA patient 38**	84	male
Histopathological features	Low-grade papillary urothelial carcinoma not infiltrating the lamina propria	
Pathological stage	pT1aG1NxMx AJCC 2017 VIII ed.	
**BLCA patient 39**	70	female
Histopathological features	High-grade urothelial carcinoma with infiltration of the submucosal connective tissue	
Pathological stage	pT1aG3NxMx AJCC 2017 VIII ed.	
**BLCA patient 40**	74	male
Histopathological features	–	
Pathological stage	–	
**BLCA patient 41**	58	male
Histopathological features	–	
Pathological stage	–	
**BLCA patient 42**	87	male
Histopathological features	High-grade papillary urothelial carcinoma infiltrating the submucosal connective tissue, without evidence of muscularis propria invasion	
Pathological stage	pT1bG4NxMx AJCC 2017 VIII ed.	
**BLCA patient 43**	58	male
Histopathological features	Low-grade papillary urothelial carcinoma not infiltrating the lamina propria	
Pathological stage	pT1aG1NxMx AJCC 2017 VIII ed.	
**BLCA patient 44**	69	male
Histopathological features	Low-grade urothelial carcinoma, non-invasive; evaluable muscularis propria is free of neoplasia	
Pathological stage	pT1a G1NxMx AJCC 2017 VIII ed.	
**BLCA patient 45**	76	male
Histopathological features	Low-grade urothelial carcinoma with initial infiltration of the lamina propria; underlying muscularis propria is free of neoplasia	
Pathological stage	pT1a G1NxMx AJCC 2017 VIII ed.	
**BLCA patient 46**	86	male
Histopathological features	–	
Pathological stage	–	
**BLCA patient 47**	64	male
Histopathological features	High-grade papillary urothelial carcinoma with initial infiltration of the submucosal connective tissue; evaluable muscularis propria is free of neoplasia	
Pathological stage	pT1aG3NxMx AJCC 2017 VIII ed.	
**BLCA patient 48**	78	male
Histopathological features	Low-grade papillary urothelial carcinoma with initial infiltration of the underlying submucosal connective tissue; all muscularis propria fragments present are free of neoplasia	
Pathological stage	pT1aG1NxMx AJCC 2017 VIII ed.	
**BLCA patient 49**	66	male
Histopathological features	Low-grade papillary urothelial carcinoma (WHO 2022) not infiltrating the muscularis propria in the evaluable samples	
Pathological stage	pT1aG1NxMx AJCC 2017 VIII ed.	
**BLCA patient 50**	74	male
Histopathological features	High-grade urothelial carcinoma infiltrating the bladder wall throughout its full thickness and invading the prostatic parenchyma	
Pathological stage	pT4aG3NxMx AJCC 2017 VIII ed.	
**BLCA patient 51**	62	male
Histopathological features	–	
Pathological stage	–	
**BLCA patient 52**	71	female
Histopathological features	Low-grade papillary urothelial carcinoma not infiltrating the lamina propria	
Pathological stage	pT1G1NxMx AJCC 2017 VIII ed.	
**BLCA patient 53**	75	male
Histopathological features	High-grade urothelial carcinoma with nests and cords growth pattern infiltrating the muscularis propria	
Pathological stage	pT2G3NxMx AICC 2017 VIII ed.	
**BLCA patient 54**	70	female
Histopathological features	High-grade papillary urothelial carcinoma (G3, WHO 2022) infiltrating the muscularis propria throughout its full thickness	
Pathological stage	pT2G3NxMx AICC 2017 VIII ed.	

### 2.2 Serum T3, T4 and TSH measurements

Blood samples were collected in 1.5 mL tubes without anticoagulant and processed as previously reported [21,22]. Briefly, after clot formation, the samples were centrifuged, and the recovered serum was stored at −20 °C until the measurement. TSH levels were determined on serum samples by a specific mouse TSH ELISA kit (Elabscience Biotechnology Inc., Houston, Texas, US). Total T4 and T3 were measured by the ADVIA Centaur XP Immunoassay system using a commercial kit, as recommended by the manufacturer (Siemens Healthcare Diagnostics, Camberley, UK).

### 2.3 Extraction of cfRNA from plasma

cfRNA was isolated from human plasma using TRIzol™ reagent (Life Technologies Ltd, Carlsbad, California, USA, Catalog number: 15596018) following a modified phenol-chloroform extraction protocol optimized for low-abundance circulating RNA. All procedures were performed using RNase-free consumables and under sterile conditions to prevent RNA degradation. Plasma samples (500 μL) were thawed on ice and briefly centrifuged at 3.000×*g* for 10 min at 4 °C to remove residual cellular debris. Only non-hemolyzed plasma was used for downstream analysis. Two volumes of TRIzol™ were added to each plasma sample. Samples were vortexed for 15–30 s and incubated for 5 min at Room Temperature (RT) to ensure complete lysis. 0.2 vol of chloroform were added to each tube, followed by vigorous shaking for 15–30 s. Tubes were incubated for 3 min at RT and centrifuged at 12.000×*g* for 15 min at 4 °C. The aqueous phase was carefully transferred to a new RNase-free tube. To maximize yield of low-abundance cell-free RNA, 2.0 μL glycogen (Life Technologies Ltd, Carlsbad, California, USA, Catalog number: R0561) were added as carrier and 2.5 vol of ice-cold 100 % ethanol. Samples were mixed gently and incubated at −80 °C overnight to precipitate RNA. RNA was pelleted by centrifugation at 15.000×*g* for 20 min at 4 °C. Pellets were washed with 500 μL of 75 % ethanol and centrifuged again at 7.500×*g* for 5 min. After removing the ethanol, the RNA pellets were air-dried for 5 min. RNA samples were resuspended in 10 μL RNase-free water and incubated at 55 °C for 5 min to enhance solubilization. RNA concentration was measured using NanoDrop spectrophotometry.

### 2.4 Reverse transcription and qRT-PCR workflow

Complementary DNA (cDNA) was generated from 2.0 μg of total RNA using the SuperScript™ VILO™ MasterMix (Life Technologies Ltd., Carlsbad, CA, USA, Catalog number: 11755-050), following the reaction setup recommended by the supplier. Quantitative Real-Time PCR (qRT-PCR) analyses were performed on a CFX Connect Real-Time PCR Detection System (Bio-Rad, Hercules, CA, USA, Catalog number: 1855201) using SYBR Green as intercalating fluorophore (BioRad, Hercules, California, USA, Catalog number: 1708882). Each sample was run in technical duplicate, and gene expression values were normalized to the endogenous reference gene Cyclophilin-A. Relative expression levels were calculated using the comparative Ct approach, expressing fold changes as 2^^(ΔCt_sample−ΔCt_calibrator).^ Primer pairs were designed to operate under a unified cycling program (initial denaturation at 95 °C for 10 min, followed by 40 cycles at 95 °C for 15 s and 60 °C for 1 min) yielding amplicons of approximately 200–300 bp. When feasible, primers were positioned across exon-exon boundaries to minimize amplification of genomic DNA. Primer sequences are reported in Table 3.

**Table 3:** List of oligonucleotides used for qRT-PCR.

Oligo	Name/Gene ID	Sense	Sequence
Cyclophilin A	CYPA	Forward	AGTCCATCTATGGGGAGAAATTTG
		Reverse	GCCTCCACAATATTCATGCCTTC
CyA amplicon length	195 bp		
https://www.ncbi.nlm.nih.gov/datasets/gene/5478/			
D2	DIO2	Forward	CTCTATGACTCGGTCATTCTGC
		Reverse	TGTCACCTCCTTCTGTACTGG
D2 amplicon length	211 bp		
https://www.ncbi.nlm.nih.gov/datasets/gene/1734/			
E-Cadherin	CDH1	Forward	GGCGCCACCTCGAGAGA
		Reverse	TGTCGACCGGTGCAATCTT
E-Cadherin amplicon length	64 bp		
https://www.ncbi.nlm.nih.gov/datasets/gene/999/			
GATA3	GATA3	Forward	AGTACAGCTCCGGACTCTTC
		Reverse	CTTAATGAGGGGCCGGTTCT
GATA3 amplicon length	224 bp		
https://www.ncbi.nlm.nih.gov/datasets/gene/2625/			
N-Cadherin	CDH2	Forward	ACAGTGGCCACCTACAAAGG
		Reverse	CCGAGATGGGGTTGATAATG
N-Cadherin amplicon length	201 bp		
https://www.ncbi.nlm.nih.gov/datasets/gene/1000/			
UPK3A	UPK3A	Forward	GGTCAATATGTCCACGGGCT
		Reverse	GCCAGCAAAACCCACAAGTA
UPK3A amplicon length	181 bp		
https://www.ncbi.nlm.nih.gov/datasets/gene/7380/			
Vimentin	VIM	Forward	GAACCTGCAGGAGGCAGAAG
		Reverse	CATCTTAACATTGAGCAGGTC
Vimentin amplicon length	328 bp		
https://www.ncbi.nlm.nih.gov/gene/7431			

## 3 Results

### 3.1 D2 is upregulated in bladder cancer across demographics, stages and subtypes

**Fig. 1:**
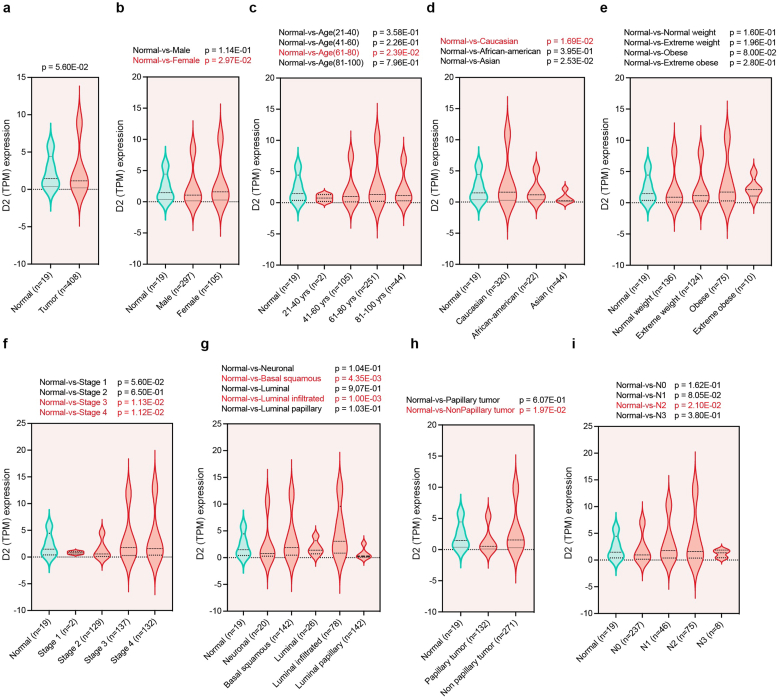
In-depth TCGA D2 expression analyses in BLadder CAncer (BLCA). a-i, Differential D2 expression analysis between normal and BLCA samples (a), stratified in function of patient's gender (b), patient's age (c), patient's race (d), patient's weight (e), individual cancer stages (f), BLCA molecular subtypes (g), BLCA histologic subtype (h) and presence and/or absence of nodal metastasis status (N0, no regional lymph node metastasis; N1, Metastases in 1–3 axillary lymph nodes; N2, Metastases in 4–9 axillary lymph nodes; N3, Metastases in 10 or more axillary lymph nodes) through the online UALCAN Database (https://ualcan.path.uab.edu). D2 levels, expressed as Transcripts Per Million (TPM), are shown using violin plots.

To characterize D2 expression and its clinicopathological relevance in BLCA, we queried the UALCAN Database (The University of ALabama at Birmingham CANcer data analysis Portal, https://ualcan.path.u ab.edu) [20] which allowed us to compare D2 levels between normal urothelium and primary BLCA samples and to assess D2 expression patterns across tumor stages and clinical subgroups. We observed that D2 was overexpressed in BLCA tumor tissues (Fig. 1a) and enhanced independently of patient's gender (Fig. 1b), age (Fig. 1c), race (Fig. 1d), weight (Fig. 1e), individual cancer stages (Fig. 1f), BLCA molecular subtypes (Fig. 1g), BLCA histologic subtype (Fig. 1h) and presence and/or absence of nodal metastasis status (Fig. 1i). Overall, these data demonstrate that D2 overexpression is a consistent molecular trait of BLCA and is maintained across patient demographics, tumor stages and pathological subtypes.

### 3.2 Patient characteristics

A total of 108 individuals were enrolled in this study, as summarized in Table 1, Table 2. The cohort included 54 healthy controls and 54 BLCA patients. In both groups, 43 participants (80 %) were male and 11 (20 %) were female. The mean age of healthy controls was 62.8 ± 15.0 years (range 22–86), whereas the BLCA group had a mean age of 67.6 ± 12.6 years. All enrolled subjects were Caucasian (100 %).

### 3.3 D2 is detectable in circulating plasma transcripts from bladder cancer patients and parallels BLCA- and EMT-related transcriptional changes

**Fig. 2:**
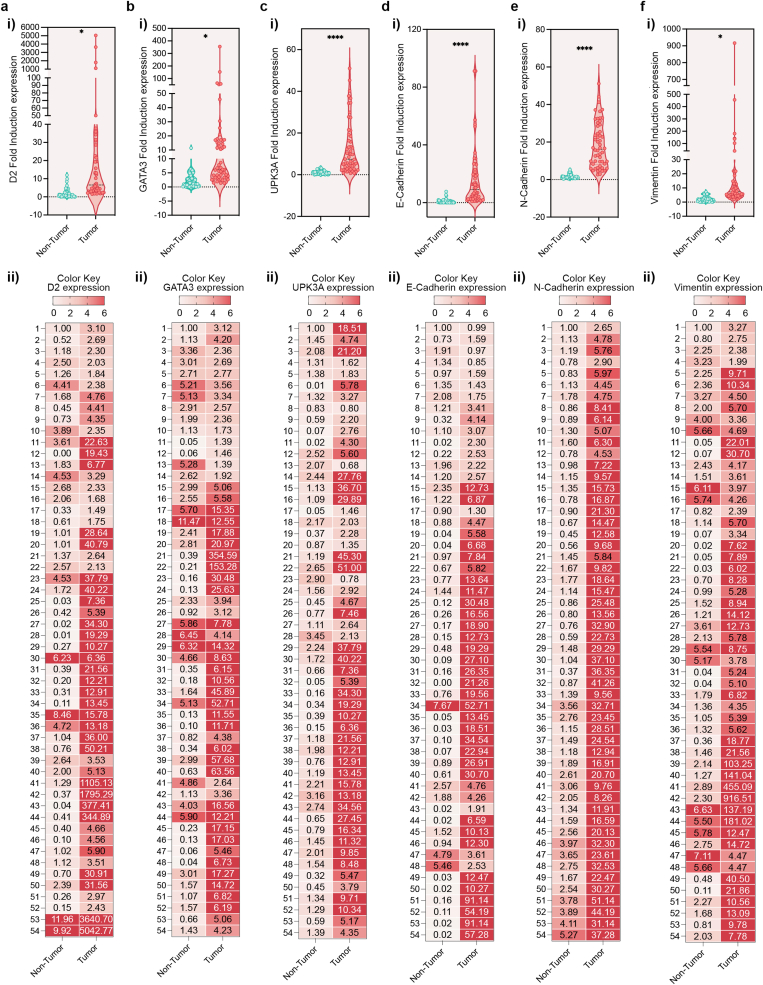
D2 is detectable and markedly overexpressed in plasma from BLCA patients compared to healthy non-tumor controls. Cell free mRNA expression of D2 (a), GATA3 (b), UPK3A (c), E-Cadherin (d), N-Cadherin (e) and Vimentin (f) assessed by qRT-PCR in plasma samples from healthy control group (Non-Tumor) and BLCA patients (Tumor). p-values were determined by unpaired two-tailed Student's t-test (∗p < 0.05; ∗∗∗∗p < 0.0001). D2 levels are shown using violin plots (upper part, i) and heatmap of raw qRT-PCR expression values (bottom part, ii) for the target gene across non-tumoral control samples and tumoral samples. Each row corresponds to the measured expression value (raw Fold Induction-derived quantity) for the gene of interest, and each column represents the two individual groups. Color intensity reflects the relative magnitude of expression, with higher values shown in more intense tones and lower values in lighter tones (Color Key gene Expression map). The heatmap allows direct visual comparison of the expression distribution between the non-tumoral and tumoral groups without transformation.

This explorative study investigates whether D2 can serve as a circulating biomarker for the early detection of bladder cancer (BLCA). mRNA levels were quantified by qRT-PCR in plasma samples collected from 54 BLCA patients and 54 healthy control volunteers to determine whether circulating tumor-derived transcripts can reliably capture D2 expression alterations. D2 plasma levels were consistently higher in BLCA patients compared with healthy controls (Fig. 2a), with the mean difference reaching statistical significance (p < 0.05). A similar trend was observed for GATA3 [18,20,23] (p < 0.05) and UPK3A [19,20] (Fig. 2b and c), two well established BLCA-associated markers, supporting the presence of tumor-related transcriptional signatures in circulation. In addition, plasma transcripts levels of both epithelial (E-Cadherin, Fig. 2d) and mesenchymal (N-Cadherin and Vimentin, Fig. 2e and f) markers were likewise elevated in BLCA samples. Rather than indicating a full loss of epithelial traits, this pattern is consistent with the coexistence of epithelial and mesenchymal-like tumor subpopulations and with hybrid epithelial-mesenchymal states, in line with the concept of partial Epithelial-to-Mesenchymal Transition (pEMT) and cellular plasticity in BLCA [[24], [25], [26], [27]]. Taken together, these results indicate that circulating D2 alterations, consistent with BLCA- and EMT-associated transcriptomic changes, reflect underlying tumor molecular features, supporting the feasibility of D2 as a minimally invasive plasma biomarker for BLCA detection and molecular characterization.

### 3.4 Circulating thyroid hormone levels remain within normal range in BLCA patients

To evaluate whether the increase in circulating D2 was associated with systemic thyroid dysfunction, serum free T3 (fT3), free T4 (fT4) and Thyroid-Stimulating Hormone (TSH) levels were measured in a subset of BLCA patients (n = 30). As indicated in Table 4, all parameters remained within their respective physiological ranges. These findings indicate that the observed elevation in circulating D2 occurs independently of systemic TH alterations and does not reflect overt endocrine dysregulation.

**Table 4:** Clinical chemistry analysis of thyroid function (fT3, fT4, TSH) in BLCA patients.

		fT3 (pg/mL)	fT4 (ng/dL)	TSH (μU/mL)
	**BLCA patient 1**	3.92	1.41	1.73
	**BLCA patient 2**	3.86	1.19	1.48
	**BLCA patient 3**	2.27	1.10	1.09
**Reference Ranges**	**BLCA patient 4**	3.10	1.54	1.28
**fT3 (pg/mL)**	**BLCA patient 5**	2.72	1.15	1.16
2.30–4.20	**BLCA patient 6**	2.61	0.76^a^	2.15
	**BLCA patient 7**	4.83^a^	1.15	0.92
**fT4 (ng/dL)**	**BLCA patient 8**	2.83	1.28	0.99
0.89–1.76	**BLCA patient 9**	2.24	1.53	0.75
	**BLCA patient 10**	3.19	1.14	1.53
**THS (μU/mL)**	**BLCA patient 11**	4.14	1.19	5.61^a^
0.55–4.78	**BLCA patient 12**	3.33	1.25	0.76
	**BLCA patient 13**	2.24	0.70^a^	0.64
	**BLCA patient 14**	2.02	0.79^a^	2.23
	**BLCA patient 15**	2.49	0.90	0.82
	**BLCA patient 16**	2.32	0.99	0.90
**Mean ± SD fT3**	**BLCA patient 17**	2.83	0.89	4.52
**in BLCA patients**	**BLCA patient 18**	1.90^a^	1.20	1.32
3.0 ± 0.74	**BLCA patient 19**	4.60^a^	2.69^a^	0.27^a^
	**BLCA patient 20**	2.60	1.16	0.54^a^
**Mean ± SD fT4**	**BLCA patient 21**	2.30	1.10	0.71
**in BLCA patients**	**BLCA patient 22**	2.70	1.04	0.70
1.2 ± 0.36	**BLCA patient 23**	2.70	1.18	0.84
	**BLCA patient 24**	3.50	0.99	1.45
**Mean ± SD TSH**	**BLCA patient 25**	3.00	1.32	1.65
**in BLCA patients**	**BLCA patient 26**	3.60	1.51	0.71
1.4 ± 1.13	**BLCA patient 27**	3.60	1.61	0.28
	**BLCA patient 28**	3.40	1.31	1.12
	**BLCA patient 29**	3.30	1.23	1.81
	**BLCA patient 30**	2.60	1.36	1.63

a The asterisks indicate parameters that fall outside the measurement range.

### 3.5 Circulating D2 plasma levels vary independently from established urothelial markers

**Fig. 3:**
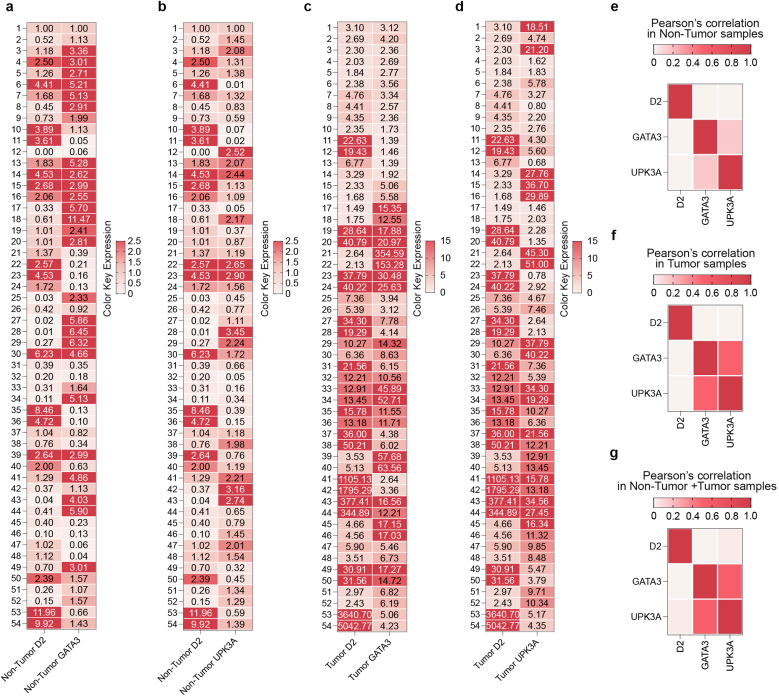
Circulating D2 levels display distinct expression dynamics compared to classical urothelial markers. Heatmaps of raw qRT-PCR expression values showing the pairwise expression patterns of D2 with GATA3 (a) and D2 with UPK3A (b) in plasma samples from the non-tumoral control group. Corresponding heatmaps depicting the pairwise expression patterns of D2 with GATA3 (c) and D2 with UPK3A (d) are shown for plasma samples from the BLCA tumor group. Each row corresponds to the measured expression value (raw Fold Induction-derived quantity) for an individual sample, and each column represents the two genes analyzed in each pair. Color intensity reflects relative expression magnitude, enabling direct visual comparison of gene co-expression patterns between non-tumoral and tumoral samples without transformation. (e–g) Pearson correlation matrices illustrating pairwise relationships among D2, GATA3, and UPK3A expression levels in plasma samples. Matrices are shown for (e) the non-tumor control group, (f) the tumor group, and (g) the combined tumor + non-tumor cohort. Color intensity reflects the strength and direction of the Pearson correlation coefficient (ρ), enabling visual comparison of gene–gene associations across the three datasets.

The results discussed above represent absolute fold changes in D2, GATA3 and UPK3A mRNA abundance between non-tumor and tumor samples, as colorimetrically illustrated in the heat-maps shown in Fig. 2 (Fig. 2a–c, part ii). To assess whether these expression differences also translate into coordinated transcriptional behavior, we next examined the pairwise correlation structure of these genes in non-tumor (Fig. 3a and b) and tumor (Fig. 3c and d) plasma specimens. In the non-tumor group, D2 showed no positive association with either GATA3 (Pearson correlation coefficient, rho, ρ = −0.18, Fig. 3e) or UPK3A (Pearson correlation coefficient, rho, ρ = −0.12, Fig. 3e). Similarly, in BLCA samples, D2 remained uncorrelated with both GATA3 (Pearson correlation coefficient, rho, ρ = −0.08, Fig. 3f) and UPK3A (Pearson correlation coefficient, rho, ρ = −0.09, Fig. 3f), indicating that D2 upregulation occurs independently of the classical urothelial markers. In contrast, GATA3 and UPK3A displayed a moderate positive correlation in tumor samples (Pearson correlation coefficient, rho, ρ = 0.50, Fig. 3f), as expected for established BLCA-associated transcripts. When all samples were analyzed together, the absence of correlation between D2 and the two urothelial markers persisted (D2/GATA3: Pearson correlation coefficient, rho, ρ = −0.03, Fig. 3g; D2/UPK3A: Pearson correlation coefficient, rho, ρ = 0.02, Fig. 3g), further confirming that D2 contributes an orthogonal and non-redundant signal to the circulating transcriptomic profile.

## 4 Discussion

The identification of reliable blood-based biomarkers for bladder cancer remains a major clinical need, particularly given the limitations of current diagnostic tools and the biological heterogeneity of urothelial tumors [1], [28], [29], [30], [31]. Circulating RNA signatures have recently emerged as promising indicators of tumor activity, yet most validated markers to date primarily capture urothelial lineage identity rather than broader metabolic or phenotypic states [32,33]. In this context, D2 represents a biologically distinct candidate: its upregulation has been implicated in tumor-associated metabolic rewiring, epithelial-mesenchymal plasticity, and stress-adaptation programs across several cancer types [12,[34], [35], [36]]. Whether these D2-associated pathways leave a measurable imprint in circulation, and whether such signals offer information independent from canonical urothelial markers, has remained unexplored. Our study addresses this gap by demonstrating that D2 transcripts are detectable in plasma and are significantly elevated in BLCA patients, suggesting that systemic release of D2 transcripts may reflect tumor-associated metabolic rewiring and dynamic changes in cellular state.

This metabolic and phenotypic plasticity is further supported by the circulating transcriptomic landscape observed in our cohort. In addition to the circulating D2 upregulation, we observed increased plasma levels of also transcripts encoding classical EMT-related markers, including E-Cadherin, N-Cadherin and Vimentin, in BLCA patients compared with healthy controls. The concurrent elevation of both epithelial (E-Cadherin) and mesenchymal (N-Cadherin, Vimentin) markers does not support a model of full EMT but rather suggests a heterogeneous circulating tumor-derived compartment, in which epithelial and mesenchymal-like populations, as well as hybrid epithelial-mesenchymal states, may coexist. This pattern is consistent with the concept of pEMT and well-documented cellular plasticity in BLCA [[24], [25], [26], [27]]. Nevertheless, given that plasma mRNA reflects a composite signal integrating contributions from tumor cells, tumor stroma and systemic responses, the exact cellular origin of these EMT-associated transcripts cannot be fully inferred from circulating RNA alone. Dedicated paired analyses of matched tissue and plasma specimens will be required to delineate the extent to which these circulating EMT-related signals derive directly from tumor cells versus the surrounding microenvironment.

A second key finding of this study is the absence of correlation between D2 and the classical urothelial differentiation markers GATA3 and UPK3A in both non-tumor and BLCA plasma samples. While GATA3 and UPK3A exhibited the expected coordinated behavior in tumor plasma samples, reflecting their shared urothelial lineage specificity [19,20,37,38], D2 varied independently from both transcripts across all conditions. This lack of co-variation indicates that D2 does not merely track the expression of established urothelial lineage markers but provides a distinct and non-redundant molecular signal within the circulating transcriptome. The biological interpretation of this independence is noteworthy. While GATA3 and UPK3A represent lineage markers capturing urothelial identity, D2 is mechanistically linked to thyroid hormone metabolism, oxidative stress responses, and cellular state transitions, including metabolic and phenotypic plasticity [12,[34], [35], [36]]. These pathways are increasingly recognized as contributors to tumor progression, metabolic reprogramming, and microenvironmental adaptation in multiple cancer types [11,16,[39], [40], [41]]. The elevation of circulating D2 in BLCA patients may therefore reflect tumor-associated alterations occurring along biological axes that are not captured by traditional urothelial markers. In this sense, D2 appears to represent an orthogonal readout of BLCA biology. From a biomarker perspective, the non-overlapping behavior of D2 relative to GATA3 and UPK3A is particularly valuable. Biomarkers that are highly correlated often yield redundant information, whereas markers representing independent biological processes are more likely to offer additive diagnostic or prognostic value. The independence of D2 suggests that integrating D2 with classical BLCA markers could potentially improve the sensitivity or specificity of multi-marker liquid biopsy panels beyond what is achievable with urothelial markers alone. From a clinical perspective, cfRNA-based liquid biopsy has the potential to complement current diagnostic and surveillance strategies in bladder cancer by providing minimally invasive access to tumor-associated transcriptional states. While cystoscopy and urine cytology remain central to disease management, circulating biomarkers may help refine molecular profiling, support longitudinal monitoring, and capture biological features not reflected by conventional urothelial markers. In this context, the detection of circulating D2 may offer additional information related to metabolic and phenotypic tumor plasticity, which could be integrated into multi-marker panels to improve patient stratification and follow-up. Although not intended to replace standard-of-care procedures, such approaches may contribute to reducing procedural burden and guiding personalized surveillance strategies pending further validation.

Collectively, our findings support a model in which circulating D2 reflects tumor-associated molecular alterations that complement rather than duplicate the information provided by canonical BLCA markers. This underscores the potential utility of D2 as a biologically distinct and clinically meaningful component of minimally invasive assays for BLCA detection and molecular characterization.

### 4.1 Alternative biological fluids in liquid biopsy: implications for bladder cancer

In BLCA, urine represents a particularly attractive biological fluid for liquid biopsy analyses, as it is in direct contact with the urothelium and enables continuous shedding of tumor-derived material. Urinary cfRNA and extracellular RNA have therefore shown promise for the detection of urothelial lineage markers and tumor-specific alterations. However, with respect to TH signaling, urine predominantly reflects downstream products of systemic hormone metabolism, including residual THs and deiodinated metabolites generated through hepatic and renal clearance, rather than the real-time transcriptional activity of deiodinase enzymes such as D2. Consequently, while urine-based assays may be well suited to capture local urothelial signals, plasma cfRNA analyses may be more informative for assessing deiodinase-driven regulatory programs and systemic metabolic adaptations.

### 4.2 Technical and biological challenges of cfRNA-based liquid biopsy

The management of cfRNA-based liquid biopsy remains technically challenging due to the intrinsic instability and low abundance of circulating RNA, which make analyses highly sensitive to pre-analytical variables such as blood processing, storage conditions, and RNA extraction efficiency. In addition, plasma cfRNA represents a heterogeneous mixture originating from tumor cells, normal tissues, and systemic host responses, limiting the precise attribution of transcript origin. Quantitative measurements are further influenced by low-input RT-PCR variability and the lack of fully standardized workflows across studies. For these reasons, cfRNA biomarkers are best interpreted as complementary molecular readouts rather than standalone diagnostic tools. In this framework, our findings support the feasibility of circulating D2 detection while highlighting the need for standardized and longitudinal validation studies.

## 5 Conclusion

Circulating D2 transcripts are readily detectable in plasma and significantly elevated in BLCA patients, indicating that D2 reflects tumor-associated molecular alterations accessible through liquid biopsy. Importantly, D2 varies independently from established urothelial markers such as GATA3 and UPK3A, suggesting that it provides non-redundant biological information. This orthogonality highlights D2 as a potential complementary component in multimarker assays aimed at improving non-invasive BLCA detection. Although cross-sectional and exploratory, these findings support further evaluation of D2 in larger, clinically annotated cohorts and in longitudinal designs assessing diagnostic accuracy, subtype specificity, and response to therapy.

## Author contributions

A.N., S.S., A.G.C., F.R., L.A., S.D.M and C.M. performed *in vitro* experiments. A.N. performed bioinformatic analysis, analyzed the results and provided scientific interpretations. P.C., L.S., F.D.G., R.L.R. collected blood samples and assisted in organizing and managing patient-related clinical data. R.S. and D.T. performed the clinical measurements of the thyroid parameters fT3, fT4, and TSH. F.C., M. D and C.M. designed the overall study, supervised the clinical and experimental procedures, and analyzed the results. A.N., M.D. and C.M. wrote the paper. All authors discussed the results and provided input on the manuscript.

## Funding support

This work was supported by grants from AIRC to M.D. (IG 29242), by a PRIN-2022 grant from 10.13039/501100003407MIUR awarded to M.D. (2022HB54P9), a PRIN-2022 grant awarded to A.G.C. (20223ZWCH2), and a grant awarded to Caterina Miro (C.M.) from 10.13039/100020581AIRC Foundation for Cancer Research in Italy (MFAG 30433).

## Declaration of competing interest

The authors declare that they have no known competing financial interests or personal relationships that could have appeared to influence the work reported in this paper.
